# Exploring the role of AI in classifying, analyzing, and generating case reports on assisted suicide cases: feasibility and ethical implications

**DOI:** 10.3389/frai.2023.1328865

**Published:** 2023-12-14

**Authors:** Giovanni Spitale, Gerold Schneider, Federico Germani, Nikola Biller-Andorno

**Affiliations:** ^1^Institute of Biomedical Ethics and History of Medicine, University of Zurich, Zürich, Switzerland; ^2^Department of Computational Linguistics, University of Zurich, Zürich, Switzerland

**Keywords:** AI, artificial intelligence, assisted suicide, euthanasia, ethics committee, synthetic data, case classification

## Abstract

This paper presents a study on the use of AI models for the classification of case reports on assisted suicide procedures. The database of the five Dutch regional bioethics committees was scraped to collect the 72 case reports available in English. We trained several AI models for classification according to the categories defined by the Dutch Termination of Life on Request and Assisted Suicide (Review Procedures) Act. We also conducted a related project to fine-tune an OpenAI GPT-3.5-turbo large language model for generating new fictional but plausible cases. As AI is increasingly being used for judgement, it is possible to imagine an application in decision-making regarding assisted suicide. Here we explore two arising questions: feasibility and ethics, with the aim of contributing to a critical assessment of the potential role of AI in decision-making in highly sensitive areas.

## 1 Introduction

In an age where artificial intelligence (AI) has emerged as a formidable tool in various fields and is increasingly used for judgment (Fogel and Kvedar, 2018; Niiler, 2019; Surden, 2019; Selten et al., 2023), it is imperative to explore its potential applications in domains that are sensitive and ethically challenging. This paper delves into one such realm, presenting an exploratory study that investigates the use of AI models for the classification of case reports related to assisted suicide procedures according to the standards defined by the Dutch Termination of Life on Request and Assisted Suicide (Review Procedures) Act (RTE, 2022),^1^ the generation of fictional but plausible cases with fine-tuned AI models, and their potential impact on shared decision-making processes.

Our exploratory study primarily aimed to explore how AI models can extract relevant information from unstructured text on assisted suicide cases, potentially assisting and enhancing ethics committees' functions. To do so, we employed a diverse range of AI models for the classification of case reports based on their content. Results indicate that AI can efficiently categorize case reports and facilitate ethical decision-making by identifying key patterns and precedents, enhancing discussions and decisions in the bioethics domain. Moreover, we tested the possibility to generate synthetic plausible cases using a state of the art large language model, fine-tuned specifically for this task. Results highlight the potential yet immature role of AI-generated synthetic cases in bioethics decision-making, showing that while 60% of cases were plausible, further development and human curation are needed to fully harness their benefits for further use.

Incorporating AI into the domain of bioethics introduces ethical considerations of paramount significance (Ekmekci and Arda, 2020; Skorburg et al., 2020; Sinnott-Armstrong and Skorburg, 2021; Klugman and Gerke, 2022). This study recognizes and addresses the potential challenges and ethical dilemmas associated with the increasing reliance on AI for decision-making in this sensitive field, first and foremost automation bias (Klugman and Gerke, 2022). It underscores the critical importance of maintaining a human presence “in the loop” for making critical decisions and emphasizes the need for responsible and ethically sound implementation of AI technology.

### 1.1 Assisted suicide in the Netherlands

Bioethics, a multidisciplinary field at the intersection of multiple disciplines, including (among others) medicine, philosophy, psychology, anthropology, plays a crucial role in shaping the ethical framework of medical decision-making. Within this broader context, certain medical procedures and decision-making processes present intricate ethical challenges. Given its profound implications, the topic of assisted suicide presents one of the most challenging ethical dilemmas to address: the deliberate choice to help someone end their own life, while invoking profound moral considerations, demands rigorous scrutiny and informed deliberation (Frosch and Kaplan, 1999; Dees et al., 2013). In this light, bioethics serves as the compass guiding the ethical discourse surrounding assisted suicide. In the Netherlands assisted suicide and euthanasia are disciplined by the Dutch Termination of Life on Request and Assisted Suicide (Review Procedures) Act (RTE, 2022) (see footnote 1). According to the act, euthanasia is the intentional termination of a person's life upon their request, while in physician-assisted suicide individuals self-administer medication prescribed by a doctor. These practices are permitted when carried out by a physician who complies with specific regulations (Buiting et al., 2009). After a physician performs euthanasia, they are required to notify the municipal pathologist by completing the appropriate notification form and submitting it during the post-mortem examination. The physician also provides a detailed report, which is mandatory under the Burial and Cremation Act. Failure to meet this requirement is considered an offense (RTE, 2022) (see footnote 1).

In addition to the detailed report, the physician typically includes other information, such as the patient's medical records, specialist letters, and the patient's advance directive. The municipal pathologist then sends this notification, along with the accompanying documents, to the relevant regional review committee. This committee assesses the reports and the euthanasia procedure (RTE, 2022) (see footnote 1).

If the committee determines that the physician has met all the required criteria, they inform the physician in writing, concluding the review process. However, if the committee finds that the physician did not fulfill one or more due care criteria, they are legally obliged to report their findings to the Public Prosecution Service and the Health and Youth Care Inspectorate, which then decide on the appropriate actions (RTE, 2022) (see footnote 1).

The committee's evaluation includes examining whether the physician adhered to the due care criteria outlined in the law, legislative history, case law, as well as professional standards. They assess whether the patient's request was voluntary and well-considered, the patient's suffering was unbearable with no hope of improvement, and there were no reasonable alternatives. They also consider whether the physician informed the patient, consulted an independent physician, and executed the procedure with proper medical care and attention regarding due care criteria (RTE, 2022). The committees responsible for reviewing euthanasia notifications in the Netherlands categorize them into two groups: straightforward notifications (about 95% of cases) and those that raise questions (roughly 5% of cases). Straightforward notifications are reviewed digitally, and committee members can communicate through a secure network without the need for physical meetings. However, if any issues or uncertainties arise during the digital review, the notification's status may be changed to non-straightforward. Non-straightforward notifications are always discussed at committee meetings, and whether a notification falls into the straightforward or non-straightforward category depends on the complexity of the case or the clarity of the information provided by the physician (RTE, 2022). In order to explore and critically assess the potential role of AI in decision-making in this highly sensitive area, we therefore tried to classify these case reports based on the same categories prescribed by the Dutch law, i.e.,: due care criteria complied with; acted in accordance with the due care criteria; voluntary and well-considered request; independent assessment; unbearable suffering without prospect of improvement; no reasonable alternative; exercising due medical care; straightforward case.

In acknowledging the significant legal dimensions of assisted suicide, it is crucial to recognize that the procedures and decisions within this study are deeply rooted in legal frameworks. While our approach primarily emphasizes the bioethical perspective, the intertwining of legal stipulations cannot be understated. The detailed legal processes, criteria for due care, and the stringent review mechanisms underscore the intersection of bioethics with legal considerations in assisted suicide. In this regard, our study also aligns with the emerging field of “legal tech”, wherein technology, especially AI, is leveraged to navigate, analyze, and streamline complex legal processes (McKamey, 2017; Soukupovand, 2021; Becker et al., 2023). This perspective opens up a broader dialogue, situating our research at the convergence of bioethics, law, and technological innovation, and highlights the potential of AI not only as a tool for ethical deliberation but also as an asset in understanding and operationalizing legal requirements in sensitive medical procedures.

### 1.2 Importance of classification

The use of AI-based classifiers on case reports could play a role both in the workflow of ethics committees and in research on decision making processes around assisted suicide. Filing, assessing and classifying these case reports is prescribed by law in the Dutch system, as every report needs to be reviewed by the competent Regional Bioethics Committee to ensure the case complied with the regulations. Moreover, the study of case reports plays a relevant role in the field of bioethics (Arras, 2001; Braunack-Mayer, 2001; Thacher, 2006), as they offer valuable insights into the ethical dilemmas emerging from practice, offering a window into the intricacies faced by medical professionals and the consequences of their decisions (Parker and Dickenson, 2001), particularly in the context of assisted suicide (Meisel, 1996; Nicholson, 2013; Gilbert and Boag, 2019), and providing a foundation for informed decision-making and policy development. Through proper classification, it becomes possible to identify patterns, trends, and therefore to develop empirically informed ethical considerations in assisted suicide procedures (Emanuel, 1994; Brauer et al., 2015). This process can shed light on the factors influencing medical decisions and their ethical implications (Brauer et al., 2015). It not only aids in academic research, but also (and most importantly) informs medical professionals, policymakers, and clinical bioethicists in their need for a deeper understanding of assisted suicide practices and the moral questions that surround them. However, the volume of these reports can be overwhelming, making their classification and analysis a daunting task. It is here that AI systems could be applied to enhance and streamline the classification process, offering the potential to extract valuable insights from the wealth of data available.

### 1.3 Limitations of manual classification and role of AI in bioethics

Manually analyzing and sorting a substantial volume of case reports with traditional approaches such as thematic analysis (Clarke and Braun, 2017) is a labor-intensive and time-consuming task. Human limitations, such as fatigue and potential biases, can hinder the accuracy and efficiency of classification (Spitale et al., 2023). This creates a space for innovative approaches to streamline the process, ensuring that critical insights are not lost due to the constraints of manual labor.

It is important to note that, until recent years, text data, often referred to as “unstructured data”, could not be fully harnessed for its wealth of nuanced knowledge. While texts written in natural language are often simply called “unstructured data” this is inaccurate from the perspective of a linguist. Language expresses knowledge in all its nuances; what was lacking until recently was the ability to explore these nuances automatically. While coarse topics and general sentiment could be extracted, these approaches remained imprecise. The discovery of text understood literally as data is only recent (Grimmer et al., 2022). Advances in Natural Language Processing (NLP), exemplified by models like BERT (Devlin et al., 2019) and GPT (Brown et al., 2020), have unlocked the capacity to explore textual data's richness, allowing for a more precise understanding of the intricate nuances within text. Such advances have broad implications, particularly in the field of bioethics, as they enable more accurate and comprehensive analysis of case reports (Cohen, 2023; Thirunavukarasu et al., 2023).

AI models are increasingly finding applications in various medical and bioethical domains, offering the potential to streamline processes and enhance decision-making (Skorburg et al., 2020; Thirunavukarasu et al., 2023). These technologies provide tools for automating tasks that were previously cumbersome and time-consuming, enabling medical professionals and bioethicists to focus on the ethical nuances of their work.

Using AI models for classifying case reports introduces numerous advantages: these models excel in efficiency, scalability, and the ability to process vast datasets rapidly. While not free from various forms of bias, AI bias is measurable and mitigatable (Nadeem et al., 2020; Liang et al., 2021; Liu et al., 2022). They can identify subtle patterns and trends that may elude human observers, contributing to a deeper understanding of the ethical considerations in assisted suicide procedures. AI models are therefore valuable allies in the effort to make sense of the ever-increasing volume of data with bioethical significance.

### 1.4 Synthetic case generation

Our study extends beyond classification, delving into synthetic case generation. In recent years, there has been a surge in interest regarding the use of AI-generated synthetic data to supplement case reports (Bélisle-Pipon et al., 2023; Spector-Bagdady, 2023; Victor et al., 2023). This approach holds the potential to revolutionize the training of AI models, for example by increasing the availability of non-straightforward cases, which as reported by the Dutch Regional Euthanasia Review Committees constitute only about 5% of the notifications (RTE, 2022). In order to train a model to recognize and classify them avoiding risks of overfitting, more would be needed for both training and testing. Moreover, generating synthetic data (Nikolenko, 2021) is useful because it solves privacy issues, as sensitive data can usually not be shared due to K-anonymity (Ciriani et al., 2007) problems. We therefore fine-tuned a GPT-3.5-turbo model (OpenAI, 2023) to generate synthetic case reports. These artificially generated cases, once assessed as plausible, hold promise not only for enhancing AI models' training on cases which are scarcely available and thus improving the classification results of AI systems (Chen et al., 2021), but also for serving as valuable educational tools for bioethicists, increasing the possibility to study a broader array of conflictual situations. These artificially generated cases, when designed with precision and assessed for plausibility, have the potential to complement real-world data, providing a broader, larger, on-demand, and more diverse dataset for training and analysis.

### 1.5 Data source

To conduct this exploratory study, we used the repository of case reports maintained by the Dutch regional bioethics committees (Regional Euthanasia Review Committees, 2017). These 1,252 reports, 72 of which are available in English, served as our primary data source. The utilization of this database ensured that our study had access to a wide array of cases, enriching our investigation and enhancing the representativeness of our findings. The number of cases available in the database is described in Table 1.

**Table 1:** Cases available in the Dutch Regional Ethics Committees database, organized per year and language.

**Year**	**English**	**Dutch**
2001–2011		53
2012		63
2013		107
2014		93
2015	9	82
2016	16	72
2017	15	97
2018	8	105
2019	24	102
2020		76
2021		130
2022		113
2023		87
Total	72	1,180

## 2 Methods

### 2.1 Data collection

To gather data for our research, we utilized web scraping techniques to collect case reports available in English from the database of the five Dutch regional bioethics committees. The code used for data collection and the resulting data are available via this study's repository (Spitale and Schneider, 2023). The data collection process was organized and executed in python within a Jupyter notebook (Jupyter, 2023), ensuring optimized explainability and readability of the code used.

The primary data source utilized for this study was the official website of the Dutch regional bioethics committees, specifically the section dedicated to case reports on assisted suicide procedures, which is publicly available at https://english.euthanasiecommissie.nl/judgments/. The data retrieval process was structured to encompass all subpages on this website, categorized according to the year of publication.

For each subpage, we implemented web scraping techniques to capture a set of essential information, including the following key attributes:

1. **Case ID**: a unique identifier for each case report.
2. **Title**: the title of the case report.
3. **Publication Date**: the date when the case report was published.
4. **Link:** the URL link to the specific case report.
5. **Introduction:** a brief introductory text accompanying the case report.
6. **Text:** the case report.
7. **Tags:** the relevant tags associated with the case report, which encompassed categories such as Age, Disorders, Due care criteria compliance, Judgment, Notifying physician, and Year of publication.

Upon successfully retrieving this information from the website, the resulting dataset was organized and exported as an Excel table. This structured dataset provided a foundation for the subsequent analysis and training.

It is important to note that, in this exploratory study, the focus was specifically on collecting and analyzing case reports available in English. While it would have been feasible to gather case reports written in Dutch and consider translation for analysis, the decision was made to work exclusively with the 72 cases available in English. This choice was driven by the study's scope and the avoidance of potential bias introduced by automatic translations. By concentrating on English-language cases, rather than including also machine translations of the cases available in Dutch, we aimed to ensure a consistent and unbiased analysis of the available data.

### 2.2 AI for case classification

In the pursuit of systematic classification of case reports in our dataset, a critical element of this study was the choice of document classification approaches. These models were used to predict various classes based on the multifaceted ethical and medical dimensions inherent to each case. The classes of prediction encompassed those described in the Dutch law. The classification process was designed to discern nuanced distinctions, such as whether due care criteria were complied with or not, whether medical care was exercised, and whether the case was considered straightforward or not. Classes to predict are summarized in Table 2.

**Table 2:** Classes to predict in case classification.

**Class**	**Type**
Due care criteria complied with	y/n
Acted in accordance with the due care criteria	y/n
Voluntary and well-considered request	y/n
Independent assessment	y/n
Unbearable suffering without prospect of improvement	y/n
No reasonable alternative	y/n
Straightforward case	y/n
Exercising due medical care	y/n

#### 2.2.1 Document classification with logistic regression

As a first method, we use supervised binary document classification to predict whether the committee agrees that euthanasia was justified (*yes*) or not (*no*). Document classification is one of the most versatile methods (Grimmer and Stewart, 2013) for content analysis. As mainly words are used as features, irrespective of their contexts, the approach is also often called bag-of-words classification. We use up to three words in sequence, a simple trigram approach, which means that every word, bigram and trigram is a feature, as long as they occur more than 5 times in the corpus. This gave us 3,679 features.

In the very simple algorithm of Naïve Bayes, every *feature* is given the same weight, which means that the probability of a document *text* to belong to a class *C* is calculated as follows:


(1) $$\begin{array}{l} P\left(C|text\right)=P\left(C\right)\times \underset{feature\;\in \;text}{\prod }P\left(feature|C\right) \end{array}$$

In linear or logistic regression, the training process also learns optimal weights for each feature from the training data. If linear regression were used for document classification, the formula would be:


(2) $$\begin{array}{l} P\left(C|text\right)=\overset{N}{\underset{i=0}{\sum }}w_{i}\times P\left(C|feature_{i}\right) \end{array}$$

Where *N* is the number of features and w_i_ the weight of feature *i*.

Logistic regression further maps the linear value for the predictor to a probability, using the *logit* function. For further details on linear and logistic regression for document classification, see Jurafsky and Martin (2009).

We also apply standard methods against overfitting (Dormann et al., 2013). such as a frequency threshold of 5, 10-fold cross-validation and L2 regularization. In X-fold cross validation, a model is trained X times, with X – 1/X parts used for training and evaluation on the left-out part. The left-out part has size *N* ^*^ 1/*X*, and is always different for each of the *N* runs. The final model is built from the mean of the individual *N* models. Schreiber-Gregory and Jackson (2018) explains that the gist of regularization is to add a penalty to each model parameter. The effect of this smoothing technique is that the model generalizes better to the data instead of overfitting.

Document classification uses vector space models, in which there are typically as many dimensions as features. Raw frequency or better keyword metrics like TFIDF are used to represent each document. Similarity between documents, or also between words, is expressed by the similarity of the vector, using the cosine metric. The cosine of the angle between two vectors pointing into the same direction is 1, while the cosine of a right angle is 0. For document classification, feature by document matrices are used. In our data, we thus get a matrix of 72 documents times 3,679 features, a high dimensionality that is already taxing for calculations, and has the disadvantage that it cannot profit from feature similarity, e.g., the fact that in our texts *think, assess*, and *agree*, or *illness, disease* and *condition* are very similar.

As document classification models capture linear relations between features and classes, feature weights can be interpreted as salience or keyword measures. For instance, Schneider uses the feature weights to describe the differences between Swiss High German and German High German (Schneider et al., 2018). Disadvantages of linear models are that they cannot capture complex non-linear relationships, for instance negations, multi-word units or recognize similar words.

#### 2.2.2 BERT NLI model

In order to recognize similar words, one can learn word similarity from large collection of contexts, exploiting the Firthian hypothesis (Firth, 1957). Language philosophy dating back to Wittgenstein (1958) has stipulated that words are defined in their context, and that word usage updates their meaning (Bybee, 2007). Contexts can also be calculated using vector space models, this time with word × word matrices instead of document × word or document × feature matrices as we have used them for document classification. While these, so-called distributional semantics models, perform well, they need very large amounts of texts, leading to matrices of 10,000 times 10,000 words and more, which are taxing to calculate in practice. Also, it has been shown that dimensionality reduction techniques such as SVD (Deerwester et al., 1990) do not only reduce dimensionality to more manageable values, but also add smoothing and recognize similar words, leading to a more performant input for the calculation of word similarities with vector models (Baroni and Lenci, 2010). Typically, 100–300 dimensions are used. More recently, predictive neural models have shown to perform better than vector models (Baroni et al., 2014). The corresponding representations, again typically using 100–300 dimensions, are compatible with distributional semantic models and are known as word embeddings.

While it is beyond the scope to explain neural networks, they can be thought of as an arrangement of neurons, where each neuron is a logistic regression, which either fires or not, like a neuron in the brain. In classical feed-forward networks, neurons are arranged in rows and layers, for instance five rows time five layers, leading to 25 neurons, where each row is connected to each other row in the subsequent layer. More recent neural network architectures have more complex layouts, with feedback to earlier layers (for instance RNN = recurrent neural networks) and increasingly more neurons. One successful layout type is known as transformers, a network type which manages particularly well to distinguish between relevant and irrelevant information, also across longer contexts, the mechanism is referred to as *attention* (Vaswani et al., 2023).

Like all supervised approaches, also these models learn to predict classes from large sources of training data. Typically, however, the training is not initially adapted to the task to be solved, and training data is several orders of magnitude larger, so large that the training cannot be performed on a normal desktop computer. These models are also called Large Language Models (LLMs).

As large amounts of data are key for all supervised methods, and as data availability is the bottleneck, the search for meaningful task for which almost infinite amounts of correctly annotated data is available is the prediction of the next word. This is the central task in BERT models, and also in GPT, with the difference that BERT predicts words both from left-to-right and from right-to-left. This method is also called *self-supervised*. It is supervised because a class is predicted (the next word), but it is completely data-driven like unsupervised approaches, as no external manual annotation is used. Self-supervised LLMs can be said to be models that are trained for the wrong task—unless you want to generate text automatically. These models have an excellent world knowledge but no task-specific knowledge. Task-specific knowledge can be added to them with further training instances. Such fine-tuned models are available for many tasks, such as sentiment detection, Natural Language Inference, Question Answering, etc.

We use Natural Language Inference model (NLI) available to the community on huggingface, the BART large MLNI model (Facebook, 2023). It is based on the BART-large model (Lewis et al., 2019), which has been pretrained on several billions of text from webscrapes, Wikipedia etc. It has 406 million parameters (4.06 × 10^8^). For the adaptation to NLI, it has been trained further with the 393,000 training instances of the multi-lingual NLI task *multi_NLI* by Facebook (Facebook, 2022). This model has not been adapted to the task of predicting assessment of euthanasia in any way, it is thus called a zero-shot model (Xian et al., 2020). It has only been adapted to the task of predicting which conclusions can be drawn from premises. But the model already knows enough about the world to have a relatively accurate concept of euthanasia, health, and patients, as the short dialogue with the Python Jupyter Notebook reveals, reported in Table 3. In this case, “euthanasia is” is the premise, and the probabilities for a set of given conclusions, ordered by probability, are output.

**Table 3:** Top 10 concepts related to euthanasia in the model and probability of the association between the concept and the premise “euthanasia is” (range: 0–1).

**Concept**	**Probability**
Assisted suicide	0.99
The end of life	0.98
Connected to suffering	0.98
Death	0.95
An ethical dilemma	0.91
A consequence of suffering	0.84
An ethical question	0.82
A consequence only permitted for extreme suffering	0.63
A reason for suffering	0.37
A patient	0.11

For the prediction of whether the committee thinks that euthanasia is justified, we use each report separately as a premise, and ask BART large MLI for the probabilities of the following conclusions:

- For class *yes*: “The committee agrees that euthanasia was justified”
- For class *no*: “The committee thinks that euthanasia was not justified”

We set the flag multi_label to FALSE, as this is a binary decision task, and so that the two probabilities add to 1.

Compared to GPT3 or GPT-4 (discussed in the next section) BERT models can still be run locally on normal desktop computers, at least for the application phase.

#### 2.2.3 ChatGPT with GPT-4

GPT-3 and GPT-4 are also LLMs using transformers. They have been trained on even larger amounts of data. GPT-3 has 175 billion parameters (1.75 × 100,000 millions = 10^11^), three orders of magnitude more than BART-large. Training a new GPT-3 model from scratch has been estimated to use 936 MWh (Lai et al., 2022).

The only task that GPT models have been trained for is to predict the next word. ChatGPT, which currently uses GPT-3.5 or GPT-4.0 (the letter is also known as ChatGPT-4) has been further fine-tuned to give reasonable, friendly and cautious answers. ChatGPT-4 has been shown to reach human or near-human performance in many tasks, for instance in creativity (Haase and Hanel, 2023). Ronan and Schneider (in preparation) show that ChatGPT-4 can pass an English linguistics exam (end of first semester, BA level). Concerning medical applications, Beaulieu-Jones et al. (2023) attest near-human surgery knowledge, while Liévin et al. (2023) report that ChatGPT-3.5 has “human level performance” on answering multiple-choice medical exam questions (USMLE and MedMCQA).

In comparison to BERT models, it becomes rapidly clear that its deep semantic model is superior. It offers detailed and well-argued answers, as illustrated by the answer to one report, reported below. as a prompt we used the question: “The background text X from a discussion on Euthanasia is ‘…'. Given X, does everyone agree that euthanasia was justified?” in which the quoted part (“…”) is each Euthanasia report separately. The output is reported below:

The background text X indicates there were disagreements between the medical professionals involved in this case about whether euthanasia was justified. The attending physician believed euthanasia was warranted, citing the patient's unbearable suffering and lack of prospects for improvement. However, both the psychiatrist and the neurologist involved felt there were aspects of the patient's condition, specifically psychological and emotional factors, which had not been adequately addressed and might have improved the patient's quality of life. They both saw room for more treatment, making them less supportive of the decision for euthanasia. Furthermore, the committee overseeing this case expressed concern over the speed of the process, the lack of intensive communication, and the absence of consultation with relevant specialists, and did not seem to find the physician's argument for euthanasia convincing. So, based on the information given, it is clear that there is not a consensus that euthanasia was justified in this particular case.

ChatGPT offers very flexible prompting possibilities. While it typically gives extensive answers, one can explicitly ask for short or for *yes/no* answers, or refer to the context of previous answers and questions. We use the following prompt template for the classification, in which the quoted part (“…”) is each Euthanasia report separately.

‘The background text *X* from a discussion on Euthanasia is “…”. Given *X*, does the committee agree that euthanasia was justified? Please answer just with yes or no.'

### 2.3 Fine-tuning GPT-3.5-turbo for case generation

A fundamental aspect of this study was the development of a fine-tuned AI model (OpenAI, 2023) to generate synthetic case scenarios based on the knowledge extracted from real case reports on assisted suicide procedures. The process commenced with supervised fine-tuning to tailor the GPT-3.5-Turbo model specifically for the requirements of generating authentic and plausible case scenarios. This fine-tuning phase was essential to ensure that the AI model could perform optimally within the context of the Dutch Regional Bioethics Committees data. Data and code used for the fine tuning are available via this study's repository (Spitale and Schneider, 2023).

#### 2.3.1 Training data

For the fine-tuning process, the training data was sourced from the previously scraped and categorized case reports. These case reports served as the foundation for educating the AI model on the intricacies and nuances of assisted suicide cases. According to OpenAI's (2023) documentation, fine-tuning data should be structured as follows:

- A “system” message, describing the role and the “persona” of the model;
- A “user” message, containing the prompt with the request;
- An “assistant” message, containing the completion.

#### 2.3.2 System message—role and persona

To guide the fine-tuning process effectively, we defined a clear and specific role for the model:

“You are an AI assistant with expertise derived from extensive analysis of Dutch Regional Bioethics Committee data. Your primary function is to generate authentic and plausible scenarios involving requests for assisted suicide and the subsequent judgments. These case scenarios are intended to serve as valuable training material for bioethicists, ensuring they closely resemble real-world situations. Your role is to craft these scenarios with a high degree of realism and ethical complexity to aid in the comprehensive training of professionals in the field of bioethics.”

This role statement provides a framework for the model's behavior and the context within which it generates new case scenarios.

#### 2.3.3 User message—input composition

The input for the fine-tuning process comprises two key components. First, a base prompt was established to set the context for the AI model. Second, the model's training data included the classes extracted from each case by the scraper during the data collection phase (as described in the Section 2.1) as “key parameters for this case report”. These classes represented the various ethical and medical dimensions of the case and provided the model with the specific context for generating case scenarios. The base prompt is detailed below:

“We will use the following parameters to generate a case report. This report will cover two main aspects:
Part 1: Request for Assisted Suicide
In this section, we will describe a case that involves a request for assisted suicide. The information to be included comprises the patient's age, their specific medical disorders, the due care criteria, the type of judgment involved, and the identity of the notifying physician. We will detail the case, the patient's medical condition, the diagnosis, and the underlying reasons for requesting assisted suicide.
Part 2: Dutch Regional Euthanasia Review Committee Judgment
In the second part of the report, we will delve into how the aforementioned case was deliberated by the Dutch Regional Euthanasia Review Committee. We will provide insights into the ethical and legal considerations that were debated by the committee and, ultimately, share the outcome of their deliberation.
The key parameters for this case report are as follows:”

#### 2.3.4 Assistant message—output

The output specified in the data for the fine-tuning process was the text of the case reports gathered from the Dutch repository, as previously detailed in Section 2.1. These real-world case reports served as the source material from which the AI model was trained to produce synthetic case scenarios. The fine-tuning process played a pivotal role in refining the model's capabilities, enabling it to generate synthetic cases that closely emulated the intricate complexities and nuances present in the actual case reports—based on the variables detailed before.

After removing one of the cases from the training dataset (as each example in the fine-tuning data should not surpass 4,000 tokens, and this case was longer), the training dataset comprised a total of 246,060 tokens. The model was trained for three epochs.

By assimilating and distilling the wealth of knowledge contained within the authentic cases, the AI model became apt at crafting scenarios that authentically captured the ethical and medical dimensions inherent in the field of assisted suicide.

#### 2.3.5 Case generation

The primary goal of the case generation process was to leverage the fine-tuned model described above to generate plausible and meaningful case reports based on various combinations of parameters representing key aspects of each case.

First, we defined a range of possible values for each of the classes representing essential elements of a case. These classes encompassed aspects such as age, disorders, due care criteria, judgment, and the notifying physician. We crafted 50 random yet meaningful combinations of these defined parameters (e.g., if “age” is below 60, the notifying physician cannot be a gerontologist). These random combinations allowed us to explore a wide spectrum of possibilities while ensuring that each case was coherent and plausible.

To guide the AI model in generating the synthetic case scenarios, we used a base prompt and the lists of values for the classes described above. This prompt outlines the overarching context and expectations for each case:

“Write a case in which the regional ethics committee decides whether a patient can have access to assisted suicide based on these variables. The case should be described in about 300 - 800 words, and in any case no more than 1000.”

The prompts, completed with the random parameter combinations, were then passed to the fine-tuned model.

The input prompts and the corresponding outputs, representing the synthetic case scenarios, were saved in a pandas dataframe and subsequently exported as an Excel file. Data and code used for the fine tuning are available via this study's repository (Spitale and Schneider, 2023).

## 3 Results

### 3.1 Document classification

#### 3.1.1 Document classification with logistic regression

We first describe the results obtained when using binary Document Classification with logistic regression and 10-fold cross-validation, the method described in Section 2.2.1.

We obtain a classification accuracy of 93.1%. Precision on the class *n* is 100%, which means that all cases predicted as *n* are actually *n*. Precision of the class *y* is lower however:five of the documents predicted as *y* are actually *n*, precision of *y* is thus 51/56 = 91%. In terms of recall, recall of *y* is 100%, while recall of *n* is 11/16 = 69%. The class *y* is much bigger; it is common for machine learning approaches to predict the larger class too often, in case of doubt the risk of error is smaller if the majority class, or in statistical terms the prior probability, is predicted.

The classification accuracy of 93.1% seems very good, but it must also be considered that the dataset is skewed: there are only 16 cases of *n*, compared to 56 cases of *y*. Accordingly, a dumb algorithm which only considers the prior and always classifies *y* already obtains an accuracy of 78%. The frequently used Kappa evaluation metric measures the improvement over the prior. It is defined as


(3) $$\begin{array}{c} \kappa =\frac{p_{o}-p_{e}}{1-p_{e}} \end{array}$$

where P_e_ is the expected probability of the prior, and p_o_ the observed probability, i.e., the accuracy. The Kappa value of our model is then 69%.

#### 3.1.2 BERT NLI classification

We now describe the classification performance of the zero-shot BART NLI model that we have described in Section 2.2.2.

We used the prompt: “The background text X from a discussion on Euthanasia is ‘…'. Given X, does the committee agree that euthanasia was justified? Please answer just with yes or no.”

Its accuracy is 63/72 = 88%. Three cases that are predicted *n* are actually y, precision of class *y* is thus 53/56 = 95%. Precision of *n* is lower (like in document classification, Section 2.2.1), 10/16 = 63%. The Kappa value is 44%. This means that the classification accuracy is only a bit less than halfway between random choice with the prior and actual classification.

#### 3.1.3 Classification by ChatGPT with GPT-4

ChatGPT-4 classifies all 72 cases correctly, which corresponds to an accuracy, precision and recall of 100%. Although GPT-4 has not been fine-tuned for this task, it beats supervised bag-of-words document classification. It performs much better than the simpler zero-shot approach using a BERT model.

While this illustrates the superior deep semantics of GPT-4, we also need to consider a few points. First, the task is relatively easy for humans. The reports are written after the recommendation has been made, in order to defend it, they thus argue clearly for their case. Second, using the optimal prompt is crucial. In our initial experiments, we first used the prompt: “The background text X from a discussion on Euthanasia is ‘…'. Given X, does everyone agree that euthanasia was justified? Please answer just with yes or no.”

This prompt had a poor performance on the task, as all contested cases were answered with “no”. Fortunately, ChatGPT can also be used to find optimal prompts. Simply asking “why?” revealed a summary of the conflict and differences of opinions: by being able to answer follow-up questions and providing explanations, ChatGPT can mitigate the black box problem. Finally, ChatGPT is too calculation-intense to be used large-scale. It needs complex architecture of servers with GPUs also for the application phase if latency is an issue, and asking GPT for millions of decisions leaves a large carbon footprint.

### 3.2 Case generation

We used the fine-tuned model described before to create a dataset of 50 distinct cases. Evaluating the plausibility of these generated cases was the focus of our process. Plausibility, in this context, refers to the degree to which a case scenario aligns with realistic and coherent narratives, consistent with the ethical complexities inherent in the training dataset. The plausibility assessment, performed manually by carefully reading each case, aimed to ensure that the synthetic cases produced by the model were meaningful. Conditions for deeming a case not plausible include the discussion of situations not disciplined by the Dutch law (e.g.,: “A 15-year-old boy who suffers from Duchenne muscular dystrophy will be taken to Switzerland by his parents to undergo euthanasia.”); impossible diagnoses (e.g.,: “Assisted suicide for a terminally ill patient with complex, life-threatening but not terminal disorders”); model hallucinations (e.g.,: “It is astonishing that the link between brain death and a clinical evaluation of due care, was being unclear is the production of greenhouse gases”).

**Figure 1:**
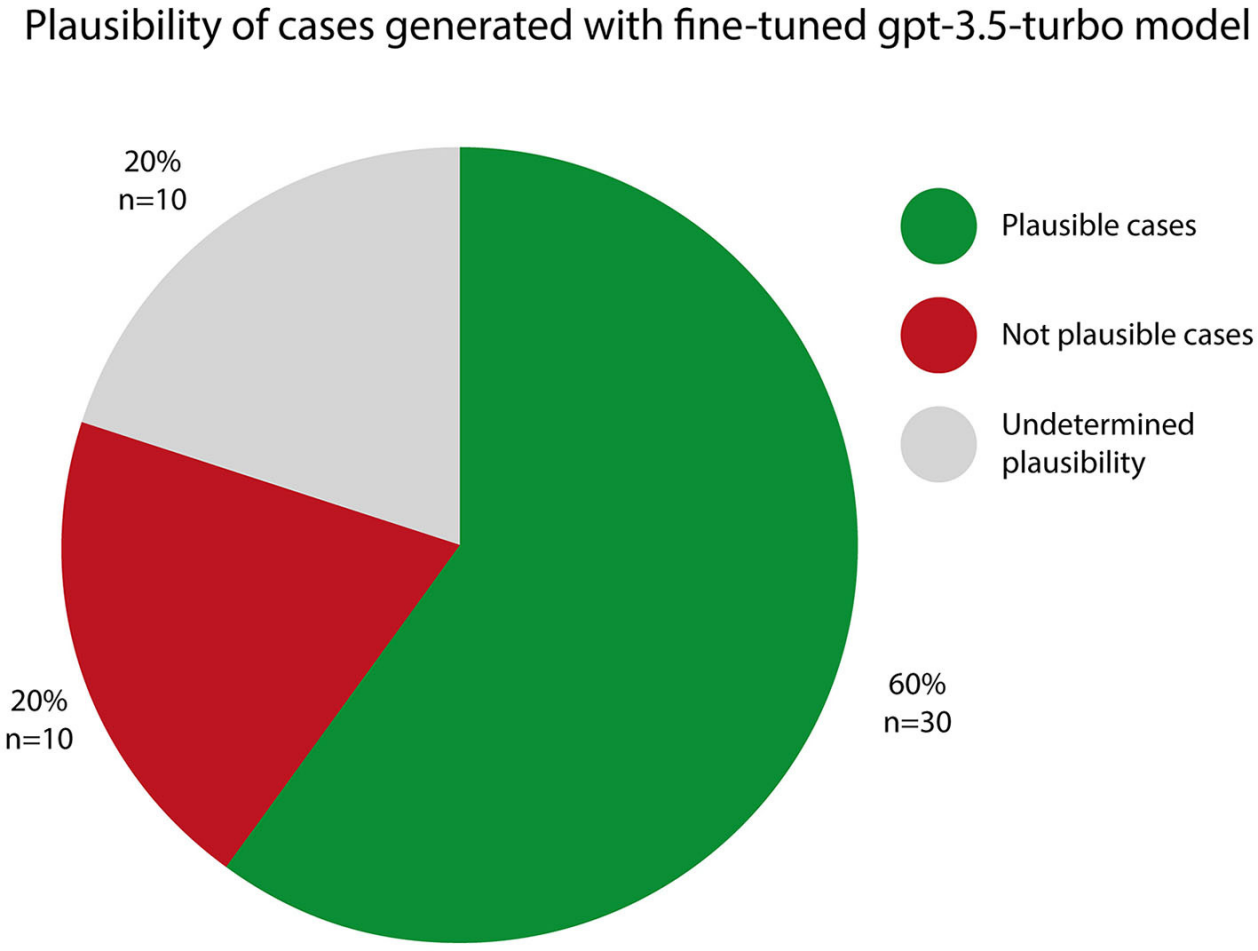
Plausibility of generated cases. Plausible Cases (60%): a total of 30 cases, representing 60% of the generated scenarios, were classified as plausible. These cases aligned well with the expectations of coherent and realistic case narratives. Not plausible cases (20%): 10 cases, or 20% of the dataset, were determined to be not plausible. These scenarios displayed inconsistencies or inaccuracies. Undetermined Plausibility (20%): in 10 cases, which accounted for 20% of the dataset, the model's output reached the token limit, preventing it from completing the case scenario. As a result, these cases were assigned “undetermined plausibility” status.

The outcome of this evaluation is detailed in Figure 1.

It is essential to note that, while these results offer valuable insights into the capabilities of the fine-tuned gpt-3.5-turbo model, there is still room for improvement. Further fine-tuning and refinement of the model could enhance its capacity to generate even more plausible and intricate case scenarios. The evaluation of plausibility remains a continuous process, striving to produce synthetic cases that closely resemble real-world situations and contribute to a deeper understanding of the ethical considerations within the realm of assisted suicide procedures in the field of bioethics.

## 4 Discussion

### 4.1 Possible uses of classifiers

It is important to emphasize that our primary goal in this exploratory study was not to develop systems able to formulate recommendations or to replace the essential function of an ethics committee. Instead, our aim was to initiate the process of understanding how specific and pertinent information related to assisted suicide cases can be effectively extracted from unstructured text.

The presented results offer valuable insights into the application of AI models for categorizing and analyzing case reports on assisted suicide cases. The results obtained in this study provide justification for a shift in the approach to case classification and feature extraction in this specific context, underscoring the potential for moving beyond the analysis of case reports containing the deliberations of ethics committees. Instead, they suggest a promising avenue for experimentation with feature extraction and case classification based on the raw case descriptions that are actively debated by the ethics committees themselves. This shift holds the potential to streamline and enhance the decision-making process by offering a proactive assessment of cases, allowing for early identification of relevant ethical considerations.

AI-based classifiers, by efficiently analyzing and categorizing case reports, have the potential to expedite the identification of pertinent precedents and patterns, functioning as knowledge repositories, and facilitating the ethical evaluation of complex cases. This capability can empower more informed and collaborative decision-making processes: in discussions among stakeholders, they can provide an automated yet informed perspective that facilitates ethical conversations, alleviate the manual workload, and contributing to more comprehensive discussions and well-informed decisions. In particular, the finding that GPT-4 used via ChatGPT correctly classified all 72 cases in our binary classification shows that the deep semantics of these systems is nearing human levels also in this task.

### 4.2 Possible uses of synthetic cases

The findings of this study, where 60% of the generated synthetic cases were deemed plausible, with an additional 20% undetermined and 20% not plausible, shed light on the promising (albeit immature) potential of synthetic cases in the realm of shared decision making within bioethics. These results, while indicative of the current state of AI model capabilities, also point to the need for further fine-tuning on larger datasets to enhance their quality and usefulness.

Synthetic cases generated by AI models have the capacity to fill a critical gap by supplementing the often-limited real-world case data, particularly in situations where access to diverse and comprehensive case reports is restricted or safeguarded due to privacy and confidentiality concerns. Once subjected to manual human curation and selection for plausibility, these synthetic cases can emerge as valuable resources.

These curated synthetic cases can be instrumental in several ways:

- Training AI models: they can serve as a resource for training AI models, allowing them to learn from a broader spectrum of simulated scenarios. For instance, it can enhance access to non-straightforward cases, which, as noted by the Dutch Regional Euthanasia Review Committees, make up only around 5% of the notifications. Utilizing synthetic cases enables the training of a model to correctly categorize real cases while mitigating the risk of overfitting, thereby contributing to the quality and accuracy of AI-based analyses.
- Research and experimentation: the availability of synthetic cases facilitates research and experimentation in a controlled and ethical manner. This is particularly essential to ensure compliance with ethical guidelines and regulations while avoiding any breeches of *k*-anonymity and risks related to the privacy and confidentiality of actual patients.
- Human Training: synthetic cases can also play a role in augmenting the training of new bioethicists, medical professionals, and other stakeholders involved in the shared decision-making process. By providing additional practice in analyzing and making decisions on various assisted suicide scenarios (which can be generated on demand, specifying the desired parameters, such as the age of the patient, the underlying condition, or the compliance with the due care criteria), they can significantly enhance the skills and judgment of bioethicists, improving the quality of shared decision making.
- Investigation of impact factors: synthetic cases offer a controlled environment for exploring the impact of specific factors or variables on ethical decision-making. This can lead to valuable insights and potential improvements in bioethics.

Despite the significant benefits, it is crucial to acknowledge the limitations of synthetic cases, as they may not fully capture the complexity and uniqueness of real cases, and they need to be checked for plausibility. Therefore, human oversight and validation remain essential to ensure the appropriateness and accuracy of the generated synthetic cases in ethical training and decision-making processes. While these tools hold great promise, the human element remains indispensable, ensuring that ethical considerations and context-specific nuances are thoroughly addressed during the shared decision-making process.

### 4.3 The hybrid approach

A hybrid approach that combines the strengths of AI models and human expertise offers a promising path forward. Rather than seeking to entirely replace human bioethicists, this approach envisions AI models as supporting tools, augmenting and assisting bioethicists in a manner that maximizes the benefits of both.

This hybrid approach ensures that AI models are not used to replace the critical human element in bioethics. Bioethicists continue to play a central role in addressing ethical nuances, contextual factors, and making the final decisions: they provide the essential ethical oversight and accountability in the decision-making process, ensuring that AI-generated classifications and insights align with ethical guidelines and regulations. Their expertise is crucial for validating and, if necessary, challenging AI-generated recommendations.

To explore the practical implications of a hybrid approach in practice, measured integration strategies should be considered. The initial step would involve gathering feedback from committee members through structured interviews or surveys. This feedback would be invaluable to better align this hybrid model with the real-world dynamics of the committees, and can help refine the approach, ensuring it meets both practical and ethical standards.

Subsequently, we suggest a tentative incorporation of AI tools in the committees' workflows, primarily for preliminary analysis of case reports. This could potentially assist in highlighting complex ethical issues for more focused human deliberation. To ensure relevance and efficacy, this approach could be illustrated through small-scale case studies, designed to further test AI's utility in identifying key ethical considerations in assisted suicide cases. This pilot would involve a gradual and closely monitored introduction of AI tools, accompanied by a robust feedback mechanism for continuous assessment and improvement. This careful and methodical approach aims to ensure that the integration of AI supports and augments the committees' essential ethical decision-making processes without overstepping its intended auxiliary role.

## 5 Conclusion

In the complex and sensitive domain discussed in this paper, the role of AI models and human bioethicists is unmistakably distinct. While the AI models showcased in this study have demonstrated significant potential, it is vital to underscore that we are far from the point where human bioethicists can be replaced by artificial intelligence. The intricacies of ethical decision-making, the need for nuanced contextual understanding, and the inherent value of human empathy and judgment remain indispensable.

However, what this exploration tried to illuminate is a path toward a plausible future in which AI models serve as assistive tools, complementing the expertise of human bioethicists. The results presented here indicate that AI models can efficiently classify, analyse, and create case reports, assist in ethical evaluations, and offer insights that contribute to more comprehensive discussions.

Future research in this field can delve into further fine-tuning AI models on larger and more diverse datasets to enhance their plausibility in generating synthetic cases, improving the quality of AI-generated insights and recommendations. Another interesting future direction is the exploration of models of collaboration between AI models and human bioethicists: understanding how these partnerships can be optimized, roles defined, and ethical oversight maintained is crucial. This is closely related to the development and refinement of ethical frameworks that guide the use of AI in bioethics, addressing issues such as transparency, accountability, and the mitigation of potential biases to ensure responsible and ethical AI applications. Finally, for the time being there are no standardized validation and benchmarking procedures for AI models in bioethics. These procedures are needed to ensure that AI recommendations and classifications meet a certain quality and ethical standard.

In conclusion, while the replacement of human bioethicists by AI models in decision making on assisted suicide requests remains distant and undesirable, we stand at the threshold of a collaborative and augmented future, where a hybrid approach presents an effective collaboration of AI models and human bioethicists. By leveraging the strengths of both, this approach ensures efficiency, accuracy, and a deeper understanding of complex ethical dilemmas, reflecting the recognition that while AI models can enhance the decision-making process, the invaluable expertise and ethical judgment of human bioethicists remain irreplaceable.

AI models, when carefully applied to the analysis and categorization of assisted suicide case reports, have significant potential to aid decision-making in this complex and sensitive field. They can enrich bioethical and legal discussions, impacting ethical practices, research, and the field of legal tech.

In conclusion, this approach promises to offer support and insights for bioethicists and legal professionals, leading to more informed and comprehensive decisions in the complex realm of ethical and legal deliberations related to assisted suicide.

## Data availability statement

The code used for data collection and the resulting data are available via this study's repository (Spitale and Schneider, 2023). It can be found here https://osf.io/7zgmj/.

## Author contributions

GSp: Conceptualization, Data curation, Investigation, Methodology, Project administration, Software, Writing—original draft, Writing—review & editing. GSc: Conceptualization, Data curation, Investigation, Methodology, Project administration, Software, Writing—original draft, Writing—review & editing. FG: Conceptualization, Validation, Writing—review & editing. NB-A: Conceptualization, Funding acquisition, Project administration, Resources, Supervision, Validation, Writing—review & editing.

## Footnotes

^1^Review procedures of termination of life on request and assisted suicide and amendment to the Penal Code and the Burial and Cremation Act. Senate, session 2000–2001, 26. 691, number 137 (2001).
